# Occurrence of *Fusarium* Species and Determination of Their Toxins From Poultry Feeds During Storage

**DOI:** 10.1155/2024/3474308

**Published:** 2024-10-15

**Authors:** Youssuf A. Gherbawy, Abdullah Altalhi, Pet Ioan, Eman G. A. M. El‐Dawy

**Affiliations:** ^1^Department of Botany and Microbiology, Faculty of Science, South Valley University, Qena, Egypt; ^2^Applied and Environmental Microbiology Center, Faculty of Science, South Valley University, Qena, Egypt; ^3^Department of Biology, College of Science, Taif University, P.O. Box 11099, Taif 21944, Saudi Arabia; ^4^Biotechnology Department, Faculty of Bioengineering and Animal Resources, University of Life Sciences “King Mihai I” From Timisoara, Calea Aradului, no. 119, Timisoara 300645, Romania

**Keywords:** deoxynivalenol, fumonisins, *Fusarium* spp., poultry feedstuffs, T-2 toxin, zeralenone

## Abstract

At a global scale, grains and poultry feeds are the primary sources of feed. Due to their considerable significance, any fungi capable of infecting these feedstuffs can pose a threat to both food safety and security. *Fusarium* spp. are a highly significant group of organisms. Fumonisins (FBs), deoxynivalenol (DON), trichothecene (T-2), and zearalenone (ZEN) are classifications of mycotoxins that are synthesized by *Fusarium* species. Their presence is associated with a range of factors that occur during growth, processing, and storage. We have recorded the high occurrence of *Fusarium* spp. in grains and poultry feeds in all tested samples. *Fusarium* (*F*) *oxysporum* was the most common species that appeared in all tested two hundred samples. FB1 was the predominant toxin that appeared with the highest concentration in 56 pellet samples with the range of 10.34–1043 *μ*g/kg. Also, it occurred with levels of 4.67–956 *μ*g/kg in the tested ingredients samples. *Fusarium verticillioides* isolates were the highest producers of FB1. *Fusarium* spp. isolates showed positive FB1 production with 84.6%, 82.5%, 82.2%, and 78.1%, isolated from pellet feed samples that were collected from Alhassa, Jeddah, Qassim, and Riyadh, respectively. 31.6%, 76.9%, 23.1%, 83.3%, and 88% of tested *Fusarium* spp. strains exhibited FB1 production in samples of barley, corn, sorghum, soybean, and wheat bran, respectively, with the range of 18–655 *μ*g/kg. Genes responsible for FB1, DON, T-2, and ZEN production were detected in the *Fusarium* spp. isolates.

## 1. Introduction

The presence of *Fusarium* fungus species on feed ingredients can result in contamination with various mycotoxins, which is influenced by factors such as temperature, substrate, and humidity. Chicken feed is a combination of different components, which increases the likelihood of many mycotoxins occurring in chicken feeds. This is because many mycotoxins can develop under comparable environmental conditions [1].

Poultry feed is produced using a variety of ingredients, including cereal grains, oilseed meal and cake (such as soybean, canola, sunflower, and rapeseed), rice bran, corn, fish meal, blood meal, bone meal, and feather meal. These ingredients are prone to contamination by mycotoxins, resulting in the presence of multiple mycotoxins in the composite feed [2]. Feeding is an important part of poultry production. Both broilers and layers are effective at converting feed to food products [3], hence there is a substantial risk of mycotoxins being carried over into edible byproducts from poultry birds given the contaminated feeds. The presence of mycotoxins in poultry feeds poses a significant risk to the health and performance of poultry. Broiler and layer chickens in farms affected by mycotoxicosis display various symptoms, including weight loss, decreased feed conversion efficiency, weakened immune system, inadequate response to vaccination, reduced fertility, increased likelihood of egg blood spots, enlarged kidneys, pale fatty liver, erosion of the gizzard, higher occurrence of leg malformations, internal bleeding, inclusion body hepatitis, and oral lesions [4]. Adverse circumstances can have a detrimental and significant impact on poultry farming [5].

Mycotoxins are metabolites synthesized by fungi. *Fusarium* species can produce many types of exotoxins, including zearalenone (ZEN), fumonisins, butenolide, and trichothecene (T-2) [6]. Class B T-2 consists of deoxynivalenol (DON), nivalenol (NIV), and their acetylated derivatives. Cereals that have been infected by *Fusarium* sp. are also polluted by the same exotoxins. Furthermore, the high toxicity of these substances poses a significant risk to the health of both humans and animals who consume the contaminated food [7, 8].

This study aimed to survey the occurrence of *Fusarium* spp. and their toxins-contaminated poultry feedstuff samples in Saudi Arabia and detect the genes associated with DON, fumonisins, T-2, and ZEN production by *Fusarium*.

## 2. Materials and Methods

### 2.1. Gathering of Poultry Feed Samples

Samples were collected from markets in four regions of Saudi Arabia (Alhassa, Jeddah, Qassim, and Riyadh). A total of two hundred samples of poultry feedstuff samples (pellets and ingredients) were stored in sterilized bags. Every sample underwent analysis to detect the presence of fungal contamination.

### 2.2. Isolation and Identification of *Fusarium* spp

The dilution-plate method was used for the isolation of *Fusarium* as described by Christensen [9]. The dichloran-rose bengal chloramphenicol agar (DRBC) medium and Czapek iprodione dichloran agar (CZID) medium were used for the isolation. Pure cultures were obtained by transferring hyphal tips to malt extract agar (MEA) for 7 days at 28°C. Isolates were maintained on MEA at 4°C and identified by macroscopic and microscopic observations using the book of Leslie and Summerell [10].

### 2.3. Extraction and Quantification of *Fusarium* Toxins by HPLC/MS Analysis

Rice media were inoculated using 3 plugs of each *Fusarium* spp. as using 3 replicates. The plugs were obtained from 7-day-old cultures of each *Fusarium* isolate on potato dextrose agar (PDA). Inoculated flasks were then incubated at room temperature (25°C–27°C) for 4 weeks with daily shaking for the first few days to permit the fungi to uniformly penetrate the rice [11]. The fungus-invaded rice was transferred to plastic trays (tray/isolate) and allowed to air dry under a ventilated hood. The dry rice was then ground to the consistency of flour using a coffee grinder. Four grams of the ground rice were suspended in 40 mL ethanol/water (80 : 20, v/v) or acetonitrile/water (80 : 20, v/v) in 250 mL Erlenmeyer flasks and extracted using a high-speed automatic shaker at 150 rpm for 2 h. The supernatants were filtered using Whatman filter paper no. 4 and the filtrates were then stored at −20°C for further use. Then, 0.5 mL was transferred into the Eppendorf tube, 0.5 mL of the buffer methanol/water 50% with 1 mL acetic acid and 0.5 mL of n-hexane were added, and pH was adjusted to 4.5. The tubes were then centrifuged at 14,000 rpm at 17°C for 10 min. Following centrifugation, the bottom layer, which contains metabolites, was extracted using a syringe and then filtered through 0.20 *μ*m micropore filters directly into tiny glass vials for HPLC analysis.

4 mL of ethanol/water or acetonitrile/water was injected through a mycotoxin column (Germany). 2 mL of the elute were evaporated to dry using a speed vacuum at 50°C and the dried sample was redissolved in 0.5 mL methanol/5 mM ammonium acetate 50/50. 10 *μ*L aliquots were injected into HPLC-mass spectrometry (HPLC/MS) for the detection. HPLC column (4 *μ*m Synergi Fusion-RP 80A; 10 cm × 2 mm dimension) was eluted with water (eluant A) and methanol (eluant B) with linear gradients of 0-1 min 15% B, 1–18 min. 10–70% B, 18–19 min. 70–98% B, 19–28 min. 98% B, 28–29 min. 15% B, and 29–39 min. 15% B at a flow rate of 0.2 mL/min. The mass spectrometer was supplied with column effluent via an electrospray ionization interface. The *Fusarium* toxins, DON, T2-toxin, ZEN, and fumonisin B standards were acquired from Sigma.

### 2.4. Molecular Identification of Mycotoxigenic Isolates of *Fusarium* Species

The DNA was extracted from 4-day-old developing fusarium mycelium that had been cultivated in 500 *μ*L of potato dextrose broth (PDB) in 2 mL Eppendorf tubes and maintained at room temperature, following the procedure outlined by Zhang, Uyemoto, and Kirkpatrick [12]. 5–10 ng of genomic DNA, one microliter of each of the primer pairs ITS-Fu-f (CAACTCCCAAACCCCTGTGA) and ITS-Fu-r (GCGACGATTACCAGTAACGA), reaction buffer (50 mM KCl, 50 mM Tris-HCl [pH 8.3], and 0.1 mg/mL bovine serum albumin), 3 mM of magnesium chloride, 200 µM of dNTP, and 2.5 U of Taq DNA polymerase (Promega, Mannheim, Germany) were included in each PCR reaction mixture. The PCR profile was run for 2 min at 95°C, then for 30 cycles at 94°C for 1 min, 54°C for 30 s, and 72°C for 1 min [13].

### 2.5. Molecular Detection of Fumonisin-Producing *Fusarium* Strains

To determine the specific isolates that produce fumonisin at the molecular level, DNA samples from several fusarium isolates were analyzed using PCR techniques. This involved utilizing specific primers, Fum5F and Fum6R, which are known to target *Fusarium* species, as stated in the study conducted by Baird et al. [14]. Each reaction contained 20 ng of *Fusarium* DNA and was carried out in quantities of 50 *μ*L. The reaction mixture consisted of a 10X buffer (Taq Polymerase Kit, Fisher Scientific, New Jersey), a solution consisting of 50 mM KCl and 10 mM Tris-HCl (pH 9); 0.2 mM of each dATP, dCTP, dGTP, and dTTP; 0.5 mM of each forward and reverse primer pairs; and 1.5 mM of MgCl_2._ The PCR program included the following temperature conditions: 95°C for 3 min, cycles with 32 times and consisted of 95°C for 1 min, 60°C for 1 min, 72°C for 3 min, and the final extension was at 72°C for 5 min. The PCR products were screened by gel electrophoresis in 2.0% agarose at 1X TAE gel and stained with ethidium bromide for photography.

### 2.6. Molecular Detection of T-2-Producing *Fusarium* Strains

For the detection of DON production by the collected *Fusarium* species (*Fusarium culmorum* and *Fusarium graminearum*) Chandler et al.'s [15] method was used. DNA was extracted using the CTAB method of Nicholson et al. [16], diluted in 1XTE, and stored at 5°C. 20 ng of fungal DNA was added to a 50 *μ*L reaction mixture for each PCR amplification. The PCR conditions used were 94°C for 2 min, followed by 35 cycles of 94°C for 30 s, 58°C for 45 s, 72°C for 30 s, then a final extension of 72°C for 5 min, and a 10°C hold. The primer pair was Tri13F (CATCATGAGACTTGTKCRAGTTTGGG) and Tri13DONR (GCTAGATCGATTGTTGCATTGAG), and 282 bp fragment from gene Tri13 was obtained. PCR results were surveyed by electrophoresis on 2% agarose gels, and ethidium bromide was added to the stain. The analysis using gel doc image analysis was used [15].

### 2.7. Molecular Detection of ZEN-Producing *Fusarium* Strains

The strains were cultivated at a temperature of 25°C on PDA for 10 days. The spores were gathered using a sterile solution of 0.1% (v/v) Tween 80 (Fisher Bioblock Scientific, Illkirch, France) and preserved at −20°C in 25% (v/v) glycerol (Fisher Bioblock Scientific, Illkirch, France) until needed. To extract DNA, fungal strains were cultivated in 250-mL Erlenmeyer flasks that contained 100 mL of PDB. The broths were contaminated with 10^6^ spores and kept at a temperature of 25°C without any movement for 4 days. The mycelium was then harvested by filtration through a 0.45 *μ*m Millipore filter frozen in liquid nitrogen and then stored at −80°C before nucleic acid extraction. A primer pair ZEA-F (5′-CTGAGAAATATCGCTACACTACCGAC-3′) and ZEA-R (5′-CCCACTCAGGTTGATTTTCGTC-3′) involved in ZEA biosynthesis in *F. graminearum* was used [17]. The PCR product appeared at 192 bp in both *F. culmorum* and *F. graminearum*. The amplification process was conducted in a 50 *μ*L reaction mixture comprising the following components: 5 *μ*L of Taq polymerase buffer (10X concentration), 1.5 *μ*L of 50 mM MgCl_2_, 1 *μ*L of dNTP (10 mM concentration for each), 1 *μ*M of each primer, 1.5 U of Taq polymerase, about 100 ng of genomic DNA, and H_2_O added to reach a final volume of 50 *μ*L. The reaction condition was initial denaturation at 94°C for 4 min, followed by 35 cycles of denaturation at 94°C for 45 s, annealing at 60°C for 45 s, and extension at 72°C for 45 s. The final extension step was at 72°C for 10 min. The amplification products were separated as mentioned above.

## 3. Results

### 3.1. *Fusarium* Associated With Pellets and Ingredients of Poultry Feedstuff Samples

Representative strains from the collected *Fusarium* spp. were detected molecularly to confirm the discriminatory power for the selected primer pairs ITS-Fu-f and ITS-Fu-r. The selected strains belonged to *Fusarium acuminatum*, *F. culmorum*, *Fusarium equiseti*, *F. graminearum*, *Fusarium incarnatum*, *Fusarium oxysporum*, *Fusarium proliferatum*, *Fusarium subglutinans*, *Fusarium solani*, and *Fusarium verticillioides*. By using this primer pair, DNA samples of each tested *Fusarium* spp. produced a PCR product (389 bp), while none of the other non-*Fusarium* DNA isolates showed any PCR product. They were subjected to sequence and deposited in the GenBank, and the accession numbers were obtained and presented in Table 1.

**Figure 1:**
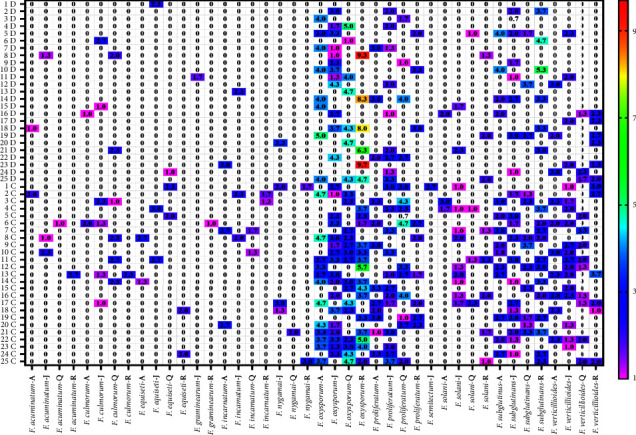
Average total counts of *Fusarium* species collected from 100 samples of pellets' poultry feedstuff and 25 samples from each city collected from Alhassa (A), Riyadh (R), Qassim (Q), and Jeddah (J), on dichloran-rose bengal chloramphenicol agar (D) medium and Czapek iprodione dichloran agar (C) medium at 27°C.

On DRBC, *Fusarium oxysporum*, *F. proliferatum*, *F. solani*, *F. subglutinans,* and *Fusarium verticilloides* were isolated from 8, 12, 7 and 5, 3, 9 4, and 3, 1, 2, 1, and 3, 4, 7, 3 and 6, 3, 4, 3 and 6 samples from pellet poultry feedstuff samples collected from Alhassa, Jeddah, Qassim, and Riyadh areas, respectively. On the other hand, they were isolated from 12, 17, 15 and 15, 9, 12, 10 and 6, 2, 9, 3 and 5, 10, 12, 9, 12 and 7, 14, 9 and 6 samples from pellet poultry feedstuff samples collected from previously mentioned areas on CZID medium, respectively. *Fusarium culmorum* was isolated from 1, 4, 4, and 1 samples from pellet feedstuff samples on a CZID medium. Also, *Fusarium graminearum* was isolated from one sample of each Riyadh and Jeddah area samples (Figure 1).

**Figure 2:**
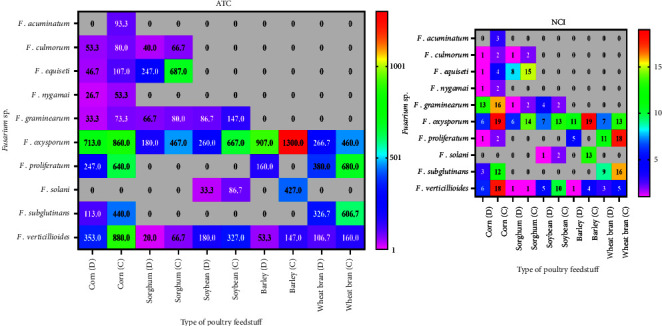
Average total counts (ATC, calculated per g of dry grain sample) and number of cases of isolation of *Fusarium* species isolated from 100 samples of corn, sorghum, soybean, barley, and wheat bran (20 samples for each type) on DRBC (D) and CZID (C) media at 27°C.

On corn grains, *F. oxysporum* and *F. verticillioides* had the highest count and frequency on the two types of media DRBC and CZID (713 and 353) and (860 and 880) colonies/g dry samples, respectively, from 20 samples collected from the four tested regions (Figure 2).

On sorghum grains, *F. equiseti* and *F. oxysporum* appeared with high counts on the two used media, by (247 and 180) and (687 and 467) colonies/g. They appeared in (8 and 15) and (6 and 14) samples, respectively. *F. oxysporum* and *F. verticillioides* were represented by (260 and 667) and (180 and 327) colonies/g samples of soybean on DRBC and CZID media (Figure 2).

*F. oxysporum* was the highest count isolated from barley grains with 907 and 1300 colonies/g samples on the two media, respectively (Figure 2).

*F. proliferatum* appeared in 11 and 18 samples with a high count of 380 and 680 colonies/g samples of wheat bran on DRBC and CZID media (Figure 2).

### 3.2. Naturally Occurring Mycotoxins in Pellet Poultry Feedstuff Samples

Mycotoxins, fumonisin (FB1), DON, T-2, and ZEN associated with pellet poultry samples were in high concentrations as compared with ingredients. FB1 was detected in 56 samples with values of 10.34–1043 *μ*g/kg. DON was recorded in 8 samples with the ranges of 240–49,380 *μ*g/kg. T-26 was estimated in 6 samples only with the lowest concentration of 35.4–48.3 *μ*g/kg. ZEN was noticed in 41 samples with the ranges of 22–57 *μ*g/kg (Table 2).

The naturally occurring mycotoxins, fumonisin (FB1), DON, T-2, and ZEN were detected in poultry feedstuff ingredients. In corn grains, FB1 was detected in 8 samples. The level of those toxins was 11.54–956 *μ*g/kg. DON and ZEN were isolated from 5 samples with levels ranging from 323 to 450 and 16 to 19 *μ*g/kg, and T-2 toxin was found in 3 samples only. From sorghum grains, ZEN and FB1 were detected with the same number of 8 samples out of 20 samples. Their levels ranged from 29 to 52 and 10.43 to 870 *μ*g/kg. The investigated mycotoxins were detected in many samples of soybean grains. Don and ZEN were detected in a range of samples from 5 to 1 and their levels ranged from 15 to 930 *μ*g/kg. From barley grains, all investigated mycotoxins were detected. T-2 toxins were not detected from the wheat bran samples, but the other toxins were detected from 2 to 8 samples of wheat bran with levels ranging from 4.67 to 36,789 *μ*g/kg (Table 2).

### 3.3. Detection of *Fusarium* Toxins From Strains Isolated From Pellet Samples Collected From Alhassa, Qassim, Jeddah, and Riyadh

Fumonisin (FB1), DON, T-2 toxin, and ZEN were tested by *Fusarium* isolates collected from the four regions of Alhassa, Qassim, Jeddah, and Riyadh.

**Figure 3:**
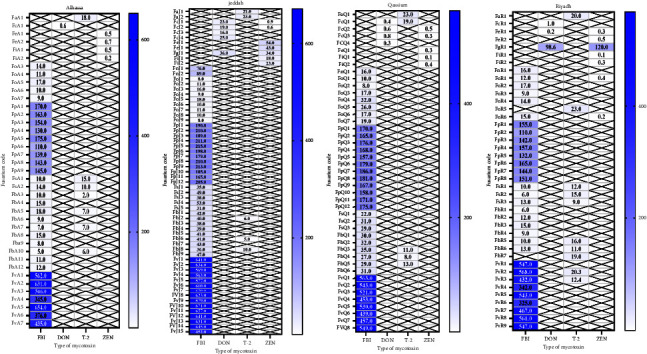
*Fusarium* toxins, fumonisin (FB1), deoxynivalenol (DON), trichothecene (T-2), and zearalenone (ZEN) produced by *Fusarium* species isolated from pellets' poultry feedstuff samples. *Fusarium* code: the second letter representing the name of the species, the third letter was Alhassa (A), Riyadh (R), Qassim (Q), and Jeddah (J), and the number was the no. of replicate of each species. Fumonisin (FB1), deoxynivalenol (DON), trichothecene (T-2), and zeralenone (ZEN).

Thirty-nine strains collected from Alhassa were tested for the four toxins. Thirty-three isolates were active producers of FB1 with a range of 5–654 PPB, and the *Fusarium verticillioides* isolates were the highest producers. One, seven, and four isolates were positive producers of DON, T-2, and ZEN with values from 0.2 to 18 PPB Figure 3.

Sixty-three isolates of isolated *Fusarium* from the Jeddah region were tested. Fifty-two strains produced FB1 with the ranges of 8–671 PPB and the highest values were noticed for *Fusarium verticillioides* isolates with 452–671 PPB. Five, another five, and seven strains were producers of DON, T-2 and ZEN toxins, respectively (Figure 3).

Forty-five strains from samples collected from the Qassim region were tested for the production of toxins. Thirty-seven isolates actively exhibited FB1 and a high concentration was observed with *Fusarium verticillioides* isolates, i.e., from 439 to 563 PPB. Four, five, and five strains produced DON, T-2, and ZEN toxins, respectively (Figure 3).

Thirty-two isolates out of 41 strains of *Fusarium*, isolated from samples collected from Riyadh, were FB1 producers. *Fusarium verticillioides* isolates showed a higher level of fumonisin and ranged from 325 to 568 PPB. On the other hand, three isolates from 41 were positive producers of DON, 10 strains actively produced T-2, and seven isolates exhibited ZEN toxin Figure 3

### 3.4. Mycotoxins Detection in Fungal Species Isolated From Ingredients' Samples

**Figure 4:**
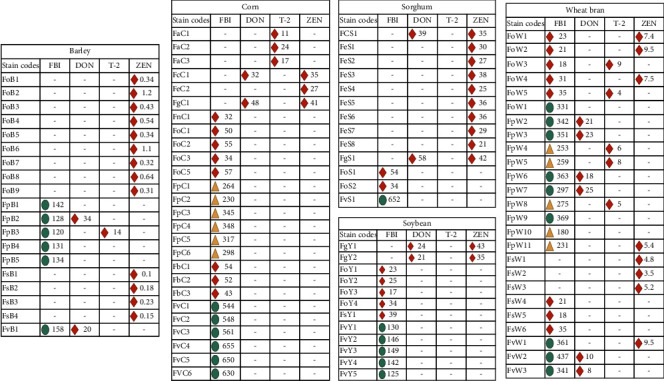
*Fusarium* toxins, fumonisin (FB1), deoxynivalenol (DON), trichothecene (T-2), and zearalenone (ZEN) produced by fungal species isolated from ingredients' feedstuff samples. *Fusarium* code: the second letter representing the name of the species, and the third letter was the name of the ingredient.

Twenty-six isolates of different *Fusarium* species isolated from corn samples were used to study their abilities to produce different *Fusarium* toxins (FB1, DON, T-2 toxin, and ZEN). Twenty isolates of different *Fusarium* species (5 species) were positive for FB1 production. Six isolates of each of *F. proliferatum* and *F. verticillioides* showed a high level of FB1 production ranging from 230 to 348 and from 544 to 655 PPB, respectively. Also, isolates of *Fusarium nygamai* (1 isolate), *F. oxysporum* (4), produced FB1 with levels ranging from 32 to 57 PPB. Single isolates of *F. culmorum* and *F. graminearum* represented by a single isolate of each species showed DON production with 32 and 48 PPB levels. Only three isolates of *Fusarium acuminatum* were able to produce T-2 toxins with levels ranging from 11 to 24 PPB. Three isolates belonging to *F. culmorum*, *F. equiseti,* and *F. graminearum* were ZEN producers with 35, 24, and 41 PPB production levels Figure 4.

Different *Fusarium* toxins were examined in 13 isolates, isolated from sorghum grains. Three out of 13 isolates were able to produce fumonisin (FB1) ranging from 54 to 652 PPB. *F. verticillioides* isolate FvS1 produced 652 PPB FB1. Only one isolate of each *Fusarium culmorum* and *F. graminearum* produced 39 and 58 PPB of DON, respectively. None of the tested isolates from different *Fusarium* were able to produce T-2. Ten isolates of *Fusarium*, *Fusarium culmorum* (1), *F. equiseti* (8), and *F. graminearum* (1) were positive producers of ZEN toxin with 21–42 PPB. Figure 4.

Different *Fusarium* toxins were examined in 12 isolates, collected from soybean grains. Twelve isolates of different fusari were used to examine their fumonisin (FB1) production. Among those isolates, *F. oxysporum* (4 isolates), *F. solani* (1), and *F. verticillioides* (5) produced FB1 ranging from 23 to 149 PPB. Two isolates of *Fusarium graminearum* were able to produce DON and ZEN ranging from 21 to 24 and from 35 to 43 PPB, respectively (Figure 4).

Nineteen isolates of different *Fusarium* species isolated from barley grains collected from Riyadh, Alhassa, Qassim, and Jeddah areas were examined for different mycotoxins' production. All *F. proliferatum* isolates (5) produced FB1 ranging from 120 to 142 PPB and a single isolate of *F. verticillioides* produced 158 PPB FB1. Two isolates, namely, FpB3 and FvB1 belonged to *F. proliferatum and F. verticillioides,* were able to produce 34 and 20 PPB of DON, respectively. One isolate of *F. proliferatum* (FpB3) produced 14 PPB of T-2. 13 isolates were able to produce ZEN. Nine and four isolates of *Fusarium oxysporum* and *F. verticillioides* produced ZEN and their production levels ranged from 0.31 to 1.2 and from 0.10 to 0.23 PPB Figure 4.

From 25 isolates of different *Fusarium* species, gathered from wheat bran samples, all *F. proliferatum* isolates (11) and *F. verticillioides* (3) isolates produced FB1 ranging from 180 to 369 and from 341 to 437 PPB. Four isolates, namely, FpW2, FpW3, FpW6, and FpW7 belonged to *F. proliferatum,* produced DON ranging from 18 to 25 PPB. From *F. verticillioides,* two isolates (FvW2 and FvW3) were able to produce 10 and 8 PPB of DON, respectively. Two isolates of *Fusarium oxysporum* and three isolates of *F. proliferatum* were T-2 toxin producers and their production ranged from 4 to 9 PPB. Three isolates of *Fusarium oxysporum*, four isolates of *F. proliferatum*, and one isolate of *F. verticillioides* were able to produce ZEN and the production levels ranged from 3.5 to 9.5 PPB Figure 4.

### 3.5. Molecular Detection of fumonisin, T-2, and ZEN-Producing Region in *Fusarium* spp

**Figure 5:**
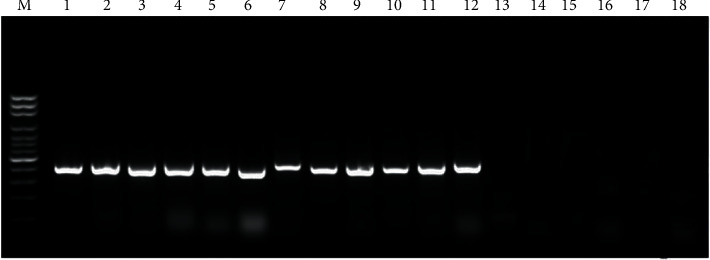
Agrose gel (1.5%) showing 419 bp-amplified products of Fum5F and Fum6R fumonisin producing regions of *Fusarium verticillioides* (lanes 1–6); *F. proliferatum* (lanes 7–12); and *F. culmorum*, *F. equiseti*, *F. graminearum*, *F. oxysporum,* and *F. subglutinans* (lanes 13–17). Lane 18: negative control (H_2_O); M: molecular-weight markers (100 bp DNA ladder, Promega).

Representative strains from the collected *Fusarium* spp. were detected molecularly to confirm their fumonisin potentials using Fum5F and Fum6R primers. Fumonisin-positive strains of *Fusarium verticillioides* and *F. proliferatum* showed PCR product (419 Bp), while none of the other *Fusarium* spp showed any PCR product (Figure 5).

**Figure 6:**
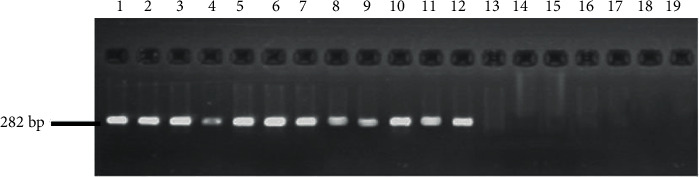
Amplification products from primer set *Tri 13* F2/*Tri* 13 DON R2 specific to deoxynivalenol chemotypes from *Fusarium culmorum* (lanes 1–9); *F. graminearum* (lanes 10–12) isolate; *Fusarium acuminatum*, *F. equiseti*, *F. oxysporum*, *F. proliferatum*, *F. subglutinans*, *F. solani*, and *F. verticillioides* (lanes 13–18). Lane 19: negative control (H_2_O).

Using *Tri 13* F2/*Tri* 13 DON R2 specific to DON chemotypes, all positive strains of *Fusarium culmorum* and *F. graminearum* showed PCR products corresponding to 282 bp. The other *Fusarium* spp. did not show any PCR product (Figure 6).

**Figure 7:**
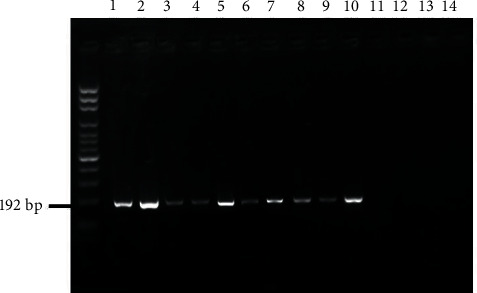
Amplification products from primer set ZEA-F/ZEA-R specific to zearalenone from *Fusarium culmorum* (lanes 1–5); *F. graminearum* (lanes 6–10) isolate; *Fusarium acuminatum*, *F. oxysporum*, *F. proliferatum*, and *F. verticillioides* (lanes 11–13). Lane 14: negative control (H_2_O).

Using (ZEA-F/ZEA-R) primer, we could easily discriminate between ZEN-producing *Fusarium* spp. and non-ZEN-producing *Fusarium* spp. The producing isolates (*F. culmorum* and *F. graminearum*) produced a PCR product of 192 bp (Figure 7).

## 4. Discussion

Feed materials may contain diverse microflora that are acquired from multiple environmental sources, including dust, soil, water, and insects at any time during growing, harvesting, processing, storage, and dispersal of the feed. Some microorganisms, primarily molds, have adapted to conditions without free water and can actively grow in stored grains. Mycobiota (molds) can also be present in feed and present a potential threat to feed quality and seed survival [18–20].

In this study, *F. oxysporum* was the predominant species isolated from both pellets and ingredients' feedstuff samples. Other species also recorded *F. culmorum*, *F. equiseti*, *F. graminearum*, *F. incarnatum*, *F. proliferatum*, *F. subglutinans*, *F. solani*, and *F. verticillioides*. From Argentinean poultry feedstuff samples, Magnoli et al. [21] indicated that *Fusarium* was isolated from 67.5% of collected samples. From the genus *Fusarium,* they isolated *F. verticillioides*, *F. nygamai*, *F. subglutinans*, *F. proliferatum*, *Fusarium semitectum*, and *F. solani*, but *F. verticillioides* and *F. nygamai* were the most frequent. From poultry feedstuff samples from Brazil, Oliveira et al. [22] showed that *Fusarium* (33.3%) was one of the most frequent genera, and the frequencies of *F. verticillioides*, *F. graminearum*, and *F. subglutinans* were 15%, 8%, and 2%, respectively. In South Africa, Naicker et al. [23] isolated *Fusarium oxysporum* from finely ground chicken feed samples with a percentage of infection reaching 40%. In India, Dass et al. [24] used PDA, DRBC, and MGA 2.5 media for isolation of different *Fusarium* species from 71 poultry feed mixture samples. Their results indicated that total counts of *Fusarium* species from poultry feed mixture samples ranged from 4.04 × 10^2^ to 1.95 × 10^5^ CFU·g^−1^. Of the 155 isolates of the genus *Fusarium*, 4 species of *Fusarium* were isolated (*F. verticillioides, Fusarium pallidoroseum*, *F. oxysporum,* and *F. solani*) and *F. verticillioides* was the most common species. The comparison between the results recorded for all *Fusarium* species on two different media, the number of cases of isolation, and the total average counts for all fusaria were higher in the CZID medium than in the DRBC medium. Bragulat et al. [25] used the CZID medium as a selective medium for *Fusarium* isolation. The same results were recorded by Mostafa and Kazem [26], who reported that a total of 365 fungi isolates were recovered from 99 maize samples collected in the six main maize productions region in Golestan, and *Fusarium* spp. were the highest (35.2%) followed *Aspergillus* sp. (2.9%), *Penicillium* sp. (1.1%), *Rhizopus* sp. (2.3%), *Mucor* sp. (1.4%), and *Alternaria* sp. (0.2%).

In this study, FB1 toxin was recorded as the highest contaminated toxin in 56 pellet samples with values of 10.34–1043 *μ*g/kg. FB1 was detected from 8 corn samples. The concentration was 11.54–956 *μ*g/kg. From sorghum grains, FB1 was detected in 8 samples out of 20 samples, with a range of 10.43–870 *μ*g/kg. In soybean grains, Don and ZEN were detected in the range of samples from 5 to 1 and their levels ranged from 15 to 930 *μ*g/kg. Wheat bran samples were contaminated with DON and the highest value was 36,789 *μ*g/kg. Ninety-one samples of maize in India were contaminated with fumonisin B1 with levels ranging from 0.1 to 87.0 ppm [27]. Sokolovic et al. [8] examined for 16 years for the fumonisins' occurrence, and more than 60% of grains and poultry feed samples were positive on FBs.

Species-specific primers were designed by Baird et al. [14] based on sequence data from the polyketide synthase (PKS) gene (FUM1-previously FUM5) responsible for fumonisin production in fungi. For selecting this primer, four sets of oligonucleotide primers were tested for their specificity using 24 strains of *F. verticillioides*, 10 strains of *F. proliferatum*, and 12 strains of other *Fusarium* species. In addition, 13 species of other fungal genera, from four phyla, were tested as negative controls. Among the four sets, primer set B consistently amplified a 419-bp fragment from the DNA in 96% of all *F. verticillioides* strains and 83% of *F. proliferatum*. All other fungi tested were negative using primer set B. A total of 38% of the *F. verticillioides* strains grown on a selective liquid medium produced fumonisin and 92% formed the toxin on a standard rice medium [14]. Karthikeyan et al. [28] found that the PKS' FUM5 region has unique primer binding sites for distinguishing fumonisin-producing *Fusarium*. Chandler et al. [15] and Yörük and Albayrak [29] found the T-2 B (DON) production by *Fusarium culmorum* and *F. graminearum* strains and positive Tri5 gene detection. Based on primers targeting the gene PKS13 involved in ZEA biosynthesis, Atoui et al. [30] developed primer pairs (ZEA-F/ZEA-R) specific to ZEN from *Fusarium*. We found the positive occurrence of this gene in *F. culmorum* and *F. graminearum.*

In conclusion, contamination of pellets' poultry feeds and grains with *Fusarium* species is a significant mycotoxicological risk. The *Fusarium* species and associated mycotoxins were identified by morphological and molecular techniques. *F. oxysporum* was the most common species that appeared in all tested two hundred samples. FB1 was the predominant toxin that appeared with the highest concentration in pellet samples, reaching 1043 *μ*g/kg. Such high occurrence of *Fusarium* and FBs may cause long-term exposure of poultry to FBs, which leads to health effects and economic decreases. To avoid the harmful effects of FBs on poultry health, it is important to take care of all the steps of production, transport, and storage of grains and pellet feeds.

## Figures and Tables

**Table 1:** Accession numbers and names of *Fusarium* species isolated from poultry feedstuff samples.

No	Accession^*∗*^ numbers	Closely related fungal sequence	Max. identity %	Given names
1	HG964349	*Fusarium acuminatum* JX114790	99	*Fusarium acuminatum*
2	HG964350	*Fusarium culmorum* DQ450880	99	*Fusarium culmorum*
3	HG964351	*Fusarium equiseti* EU326202	99	*Fusarium equiseti*
4	HG964355	*Fusarium graminearum* JX162259	99	*Fusarium graminearum*
5	HG964356	*Fusarium incarnatum* KC989098	99	*Fusarium incarnatum*
6	HG964352	*Fusarium nygamai* AY898252	99	*Fusarium nygamai*
7	HG964353	*Fusarium oxysporum* KF897851	100	*Fusarium oxysporum*
8	HG964354	*Fusarium proliferatum* KF751873	99	*Fusarium proliferatum*
9	HG964357	*Fusarium solani* KF631450	99	*Fusarium solani*
10	HG964358	*Fusarium subglutinans* AY898264	99	*Fusarium subglutinans*
11	HG964273	*Fusarium verticillioides* KC709665	99	*Fusarium verticillioides*

**Table 2:** Mycotoxin analysis (*μ*g/kg) of 100 pellets of poultry feedstuff samples and 100 samples of maize, sorghum, soya bean, barley, and wheat bran (20 samples of each), collected from Riyadh, Alhassa, Qassim, and Jeddah areas (sample no: 1–20: maize grains, 21–40: sorghum, 41–60: soya bean, 61–80: barley, and 81–100: wheat bran).

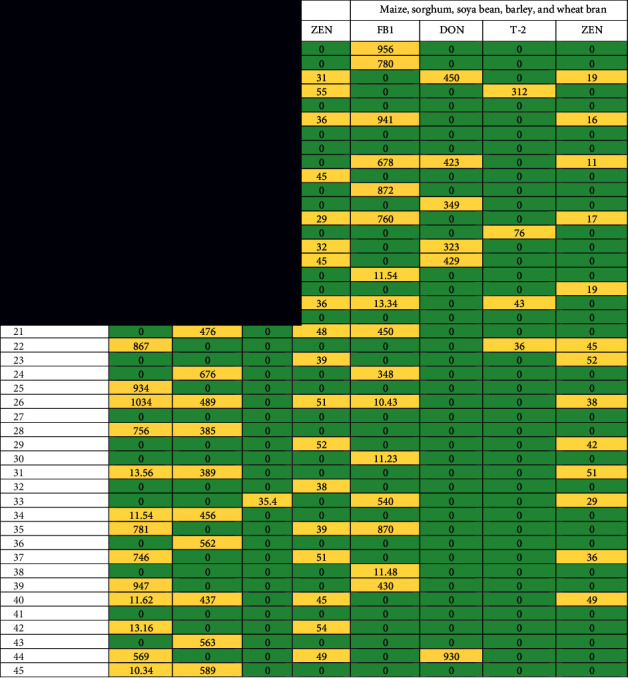
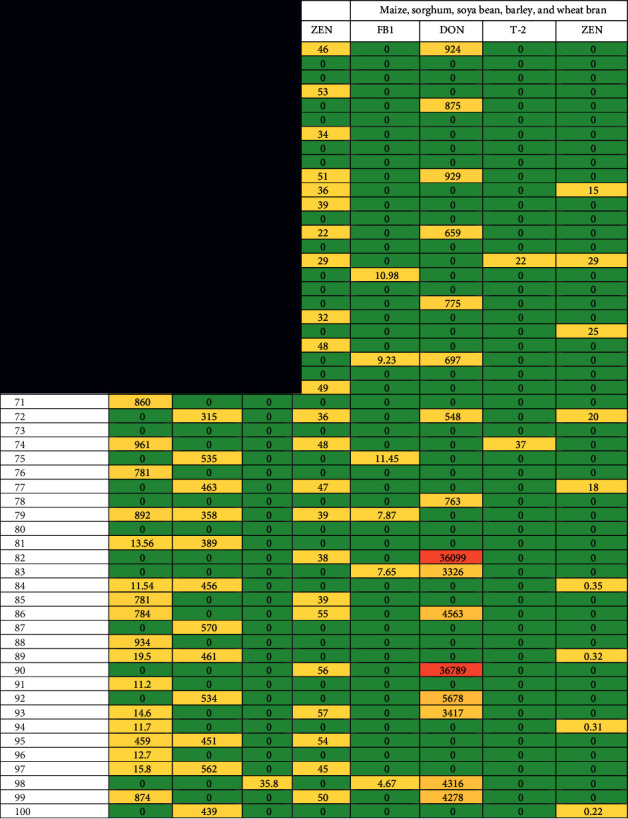

## Data Availability

All data generated or analyzed during this study are included in the manuscript.
